# Peptide Capping Agent Design for Gold (111) Facet by Molecular Simulation and Experimental Approaches

**DOI:** 10.1038/s41598-020-59144-7

**Published:** 2020-02-07

**Authors:** Che-Hsin Lin, Shin-Pon Ju, Jia-Wei Su, Dai-En Li

**Affiliations:** 1 Department of Mechanical and Electro-Mechanical Engineering, National Sun Yat-sen University, Kaohsiung, 804 Taiwan; 2 Department of Medicinal and Applied Chemistry, Kaohsiung Medical University, Kaohsiung, 807 Taiwan

**Keywords:** Atomistic models, Nanoparticles

## Abstract

The stochastic tunneling-basin hopping method (STUN-BH) was utilized to obtain the most stable peptide S7 configuration (Ac-Ser-Ser-Phe-Pro-Gln-Pro-Asn-CONH_2_) adsorbed on Au(111) facet. After the most stable S7 configuration was found, molecular dynamics (MD) simulation was conducted to investigate the thermal stability between S7 and Au facet at 300 K in both vacuum and water environment. Moreover, further design sets of peptide sequences on Au(111) facet were used to compare with S7. All molecular simulations were carried out by the large-scale atomic/molecular massively parallel simulator (LAMMPS). The Amber99sb-ILDN force field was employed for modeling the interatomic interaction of peptides, and the TIP3P water was used for the water environment. The CHARMM-METAL force field was introduced to model the S7, PF8 (Ac-Pro-Phe-Ser-Pro-Phe-Ser-Pro-Phe-CONH_2_) and FS8 (Ac-Phe-Ser-Phe-Ser-Phe-Ser-Phe-Ser-CONH_2_) interactions with Au(111). The MD simulation results demonstrate that the morphology of Pro affects the adsorption stability of Phe. Therefore, we designed two sequences, PF8 and FS8, to confirm our simulation result through experiment. The present study also develops a novel low-temperature plasma synthesis method to evaluate the facet selecting performance of the designed peptide sequences of S7, PF8, and FS8. The experimental results suggest that the reduced Au atom seed is captured with the designed peptide sequences and slowing growing under room temperature for 72 hours. The experimental results are in the excellent agreement with the simulation finding that the Pro in the designed peptide sequences plays a critical role in the facet selection for Au atom stacking.

## Introduction

Due to continuous progress in nanotechnology, nanoscale-sized catalysts with improved performance compared to their bulk counterparts have been identified. For example, the activation energy for the CO oxidation which occurs in the Langmuir–Hinshelwood (LH) reaction found on Pd nanowires is significantly lower than such a reaction progresses on Pd bulk surfaces^1–5^. In Haruta’s study^6^, the Au hemispherical nano-particles with the diameters less than 5 nm deposited on the specific metal oxides and undergoing a CO saturated hydrocarbon combustion reaction possess both high selectivity and high activity. Metal nanoparticles (NPs) that contain Au, Pt, Ag, and Pd have been found to exhibit excellent catalysis for potential oxidation, isomerization, and hydroformylation reactions^1,7^.

Besides the size effect on the catalytic efficiency, the specific NP facets may cause better catalytic performance than other factors^8,9^. Wang used SnO_2_ NPs to catalyze the oxidation of CO, and they found the catalysis performance was (111) > (221) > (101) > (110)^10^ when considering different facets. Kuzume showed that the Pt(111) facet had lower catalytic performance than that on Pt(110) for oxygen reduction in sulfuric and perchloric acid solution^11^. The aforementioned studies clearly indicate that the NP catalytic efficiency could be considerably enhanced once the fraction of facets with the better catalytic efficiency can be significantly increased.

For fabricating metal NPs with some specific facets, using a capping agent with specific molecular architecture to protect specific metal facets has been proposed^12,13^ during the NP synthesis. Numerous biological entities have been introduced to be the capping agent, including DNA, viruses, and peptides. Peptides having fine-designed amino acid sequences may allow for accurate recognition of specific metal facets as well as having the benefit of being relatively easy to prepare^14^. For PtNP, Chiu used the S7 peptide in a Ac–Ser–Ser–Phe–Pro–Gln–Pro–Asn–CONH_2_ sequence as a means to produce further growth of Pt(111) facets^12^. It is imperative to understand the peptide capping mechanism during the NP synthesis process to design peptides with diverse sequences as capping agents for different NPs with different facets. However, it is relatively challenging to observe the peptide conformations having a notably high affinity to a specific NP facet as well as the adsorption mechanisms by the direct experimental measurements. Consequently, the peptide interaction mechanism with unique metal facet by a numerical method is a feasible alternative to design the appropriate capping agent. Among all numerical methods, the molecular simulation has been widely used to investigate the interaction mechanism for the biomolecule on metal surfaces. For finding the mechanism S7 binds more strongly to Pt(111) and T7 binds more strongly to Pt(100)^15^, Ramakrishnan used the molecular dynamics (MD) simulation to study the affinity of an individual amino acid comprised of peptides S7 and T7 attached to the Pt(111) and (100) facets. For Pt(111), Phe, Gln, Asn, and Pro show a stronger binding preference, whereas Leu and Thr demonstrate stronger binding energies to the Pt(100). According to the density functional theory (DFT) calculation results^16^, Phe, Gln, Ser, and Pro displayed a higher affinity for Pt(111), while Asn, Thr, and Leu exhibited more affinity to Pt(100). An MD and experimental study by Ruan^17^ found the residue geometries, as well as their functional groups, that significantly affect the peptide interaction strength with the metal surface. For example, the aromatic six-membered ring of Phe owns a better geometrical match with Pt(111), leading to the stronger binding energy. In Oren’s MD study^18^, the formations of polypods as well as charged (or polar) groups are two main reasons amino acid residues bind with the particular NP facets. By Heinz’s MD simulation results^19^, it showed an amino acid OH group displays a stronger affinity to Pt(100); in contrast, the oxygen atom more strongly interacts with the Pt(111) surface.

Designing a peptide sequence of a capping agent for a metal NP with a distinctive facet generally involves a trial-and-error selection process^12^, which is more costly and time-consuming than computer-aided design processes. In our previous study^20^, we have successfully united the advantages of the stochastic tunneling (STUN)^21^ and basin-hopping methods^22^ as the STUN-BH method in order to predict the most stable adsorption configurations of the S7 capping agent on Pt(111). Because the lattice constant of Au (4.08 Å) is very similar to that of Pt (3.92 Å), the STUN-BH method was determined to be appropriate for use again in the current study to understand the capping mechanism of S7 on the Au(111) facet. Because the S7 peptide shows no adsorption preference on Pt(100) and Pt(110) as reported in previous studies^15,19,20^, the adsorption configurations of S7 on Au(100) and Au(110) are not considered in the current study. We also expand the simulation results into designing two new peptide capping agents, PF8 and FS8, for Au(111) facet and confirm the feasibility of these two capping agents by both simulation and experimental approaches without repeating the experimental trial-and-error process^12^.

## Material and Methods

### Simulation method

**Figure 1:**
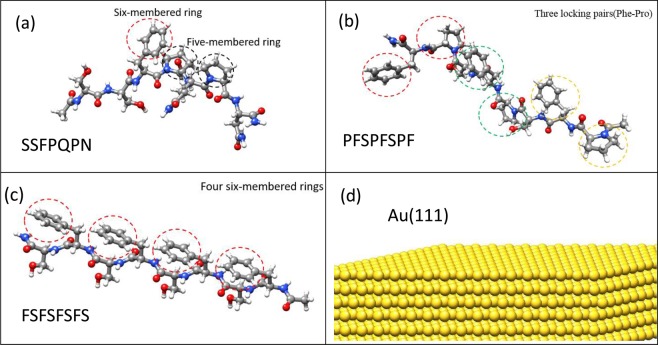
The peptides molecular structures of (**a**) conventional S7 (**b**) PF8 (**c**) FS8 and (**d**) Au(111) substrate.

Figure 1(a) shows molecular structures for S7 (Ac–Ser–Ser–Phe–Pro–Gln–Pro–Asn–CONH_2_), having one aromatic six-membered Phe ring as well as two five-membered Pro amino acid rings. After the interaction mechanism between S7 and Au(111) surface was understood, two peptides designated as PF8 and FS8 with the sequences Ac-Pro-Phe-Ser-Pro-Phe-Ser-Pro-Phe-CONH_2_ (PF8) and Ac-Phe-Ser-Phe-Ser-Phe-Ser-Phe-Ser-CONH_2_ (FS8) were designed as illustrated in Fig. 1(b,c), respectively. The detailed sequence design process for PF8 and FS8 can be seen in the results and discussion section. Figure 1(d) shows the Au (111) substrate with 6 Au layers, which were fixed during the simulation for saving the simulation time. Because the Au atoms are much heavier than C, H, and O atoms of S7 and water molecule, the Au atoms are very close to their equilibrium lattice sites during the simulation. Both the adsorption configuration of S7 on Au(111) and the interaction energy are almost identical for the fixed and unfixed Au substrate models. Consequently, the fixed Au model can completely reflect the interaction between S7 and an unfixed Au substrate model. UCSF Chimera was used to visualize the simulation results and to do the post process^23^. All molecular simulations were conducted by using the large-scale atomic/molecular massively parallel simulator (LAMMPS) package^24^. For S7, FP8, and FS8 peptides, the Amber99sb-ILDN force field^25^, which has the ability to improve the accuracy of the protein side chain interactions, was used. For the water model, the TIP3P^26^ model was used. The Amber force field has been determined to be relatively more accurate in modeling the atomic interaction of biomolecules than are other force fields such as CVFF^27^, which has been used in a previous similar topic^28^. The CHARMM-METAL force field was adopted^29^ to describe the interaction between the S7 (or PF8, FS8) and the Au(111).

In order to find the low-lying binding configurations of peptides (S7, PF8, and FS8) on the Au(111) facet, the systematical global minimum search method, STUN-BH was used. The STUN part of STUN-BH method allows the configuration to tunnel into the forbidden regions of potential energy surface (PES) by applying a nonlinear transformation to the PES. The STUN effective potential energy converts the original potential energy surface (currently for Amber99sb-ILDN and CHARMM-METAL) into a smoother potential energy surface. The effective potential energy surface still keeps the same local minimum structures as those within the original potential energy surface. The basin-hopping (BH) method by the conjugate gradient (CG) method was used to conduct the geometrical optimization for fast determining the closest local minimum peptide configuration on Au(111) in vacuum.

For each peptide (S7, PF8, and FS8), a total of 1000 independent initial conformations with different orientations to Au (111) were considered to enhance the STUN-BH domain search. Two hundred STUN-BH searches were performed for each initial peptide configuration, resulting in a total of 200,000 STUN-BH iterations that facilitate the search for the most stable peptide adsorption configuration on Au(111). For each STUN-BH iteration, the conjugate gradient (CG) optimization was conducted by following an NVT MD simulation at 500 K for 300 steps, which generates different local configurations for the next STUN-BH iteration and extends the spatial search domain by using a larger time step of 2 fs. Although each combination of φ, ψ, and χ_i dihedral angles for each peptide was not explicitly considered, the different conformations of each peptide have been included during the global minimum search process by the STUN-BH method.

Because a large amount of water molecules must be included to fill the simulation box (2,350 water molecules used in the current study) for considering the water environment, the required computational power becomes several orders higher than that in the vacuum and make the current STUN-BH method infeasible. Calculating the binding free energy by the process presented in Singam’s study^30^ for each STUN-BH iteration (a total of 200,000 STUN-BH iterations) also make infeasible the STUN-BH method applied here. In several previous docking studies^31–36^, molecular docking is first used to find the best docking configuration between the donor and acceptor by the evaluation of the scoring function or the intermolecular interaction energy. After the docking process, the complex was then used in the water environment for a long-time MD simulation to investigate the stability of the complex. After we considered previous studies using the docking method, we decide to use a similar process and adopt the interaction energy (binding energy or adsorption energy) between the peptide and Au(111) for the STUN-BH method. The binding energy E_BE_ was calculated according to the following equation:

1 $${{\rm{E}}}_{{\rm{BE}}}={{\rm{E}}}_{{\rm{peptide}}/{\rm{surface}}}-{{\rm{E}}}_{{\rm{peptide}}}-{{\rm{E}}}_{{\rm{substrate}}}$$

where E_peptide/surface_, E_peptide_, and E_substrate_ are the potential energies of peptide/surface complex, isolated peptide, and isolated Au substrate, respectively. The lower binding energy indicates the peptide is more stable on Au(111), whereas the higher binding energy indicates the peptide is less stable on Au(111). The detailed introduction on the STUN-BH search process can be found in our previous study^20^ and therefore, is not introduced.

After the STUN-BH method search, the most stable peptide configurations on Au(111) were used for the molecular dynamics simulation in the water environment. During the first 0.1 ns of all MD simulations, a spring force having spring constant of 10 Kcal/mole* Å^−1^ was applied to all peptide atoms for keeping the peptide conformation before the water molecules get equilibrated with the peptides and Au(111) surface. Next, the NVT MD was conducted at 300 K for another 4 ns in for the purpose of completely relaxing the system. The integration time step was 0.5 fs, and the Nosé−Hoover thermostat was used in order to control the system temperature. A cut-off distance of 12 Å was used to calculate the short-range electrostatics and the van der Waals interactions.

Although the STUN-BH can find the global minimum peptide configuration on Au(111) based on the interaction energy between the peptide and Au(111) in the vacuum condition, the adsorption configuration with this interaction energy does not correspond to the most probable adsorption configuration according to the adsorption free energies in the water environment as presented in Singam’s study^30^. Consequently, the global minimum adsorption peptide configuration by the current STUN-BH method is one of the low-lying adsorption configurations when the adsorption free energy was considered.

### Preparation of chemicals

Depending on the simulation results, the related experiments were conducted. The poly-peptide S7, PF8, and FS8 with the purity up to 90% were designed and synthesized by a professional biotech foundry company (Kelowna International Scientific Inc., Taiwan). The peptide solution was prepared by dissolving 2.5 mg of the peptide crystal into 20.0 mL of the ultra-pure water. Table 1 displays the detail information including the sequence of each polypeptide and the equivalent concentration of the peptide solutions. The gold salt solution was prepared by dissolving the HAuCl_4_ (Showa Chemical Industry Co., Ltd., Japan) crystal into the ultra-pure water respectively. The final concentration of the HAuCl_4_ solution was 1.0 mM. The helium gas with the purity of 4N5 (up to 99.995%) was used for the plasma generation.

**Table 1:** The detailed information about the S7, PF8, and FS8 sequences and the equivalent concentrations of peptide solutions.

	Sequence	Molecular weight (Da)	Equivalent concentration (wt%)	Equivalent concentration (mole/L)
S7	Ac-SSFPQPN-NH_2_	816.87	0.062	0.765 mM
PF8	Ac-PFSPFSPF-NH_2_	996.06		0.628 mM
FS8	Ac-FSFSFSFS-NH_2_	966.08		0.648 mM

### Setup of NP synthesis system

**Figure 2:**
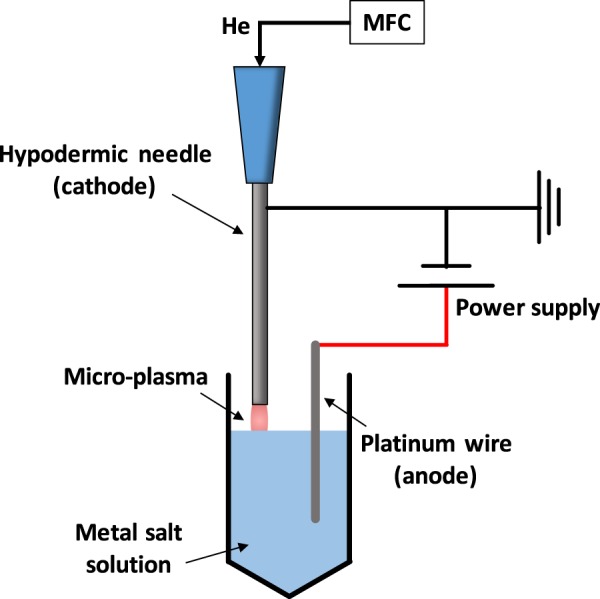
The experimental setup for synthesizing the metal nano-seed. The peptide solution was injected into the tube after the plasma treatment.

To have a slow synthesis of the metal particles by the presence of the designed peptide sequence, a home-built atmospheric plasma system was established. The AP plasma synthesis approach can reduce gold ions to form gold nanoparticles without the need of chemical reduction reagent. Moreover, the solution heating process usually used in the conventional nanoparticles reduction can be excluded from the AP plasma synthesis approach. Figure 2 shows the experimental setup for the synthesis of the metal nano-seed. The metal salt solution with a volume of 1.0 mL was firstly loaded in a 2.0 centrifugal tube (4092.6 N, DELTALAB, Spain). The helium gas with the flow rate of 50 SCCM was precisely controlled by a mass flow controller (5850E, Brooks instrument, USA), and was blown to the liquid surface through the hypodermic needle (NN-2325R, Terumo, Japan). The hypodermic needle and the platinum wire served as the cathode and the anode for the synthesis system, respectively. The high voltage power supply (230-10 R, Spellman, USA) provided the electric power (1.5 kV, 1.5 mA) for the plasma generation.

According to the theory of plasma-liquid interactions (PLIs), the hydrated electron, H^∙^ radicals and OH^∙^ radicals were generated at the interface between the plasma and the water. These chemically reactive products played the role of the reducing agent for the metal ion in the solution^37^. The metal ion in the solution was reduced and formed metal nanoparticles. A traditional reducing agent such as sodium citrate (Na_3_C_6_H_5_O_7_) and sodium borohydride (NaBH_4_) was not used in this proposed synthesis method, and the product of the PLIs such the hydrated electron, H^∙^ radicals and OH^∙^ radicals have an extremely short lifetime (10^−9^ sec. to 10^−6^ sec.)^38^. The chemical composition of the final colloidal metal synthesized by PLIs is much simpler than the colloidal metal synthesized by traditional methods such as the Turkevich method or Brust method. This study supposed that the capping performance of the peptide molecule is more evident in this chemically cleaner environment.

The helium plasma processed the metal salt solution for reducing the metal ion in the solution. According to our previous study^39^, the plasma treating time for synthesis the nanoparticles with the diameter about 10 to 20 nm was set to be 300 seconds. In this study, the metal ion solution was treated by the plasma for 30 seconds to synthesize the nano-seed with the diameter smaller than 10 nm. After plasma treatment, 1.0 mL of the peptide solution was injected into the metal salt solution. Next, this mixed solution was placed statically in a dark, 25 °C environment for 72 hours. The Au^3+^ inside the mixed solution reduced and attached on the peptide capped and free-suspended nano-seed for the spontaneously.

## Results and Discussion

**Figure 3:**
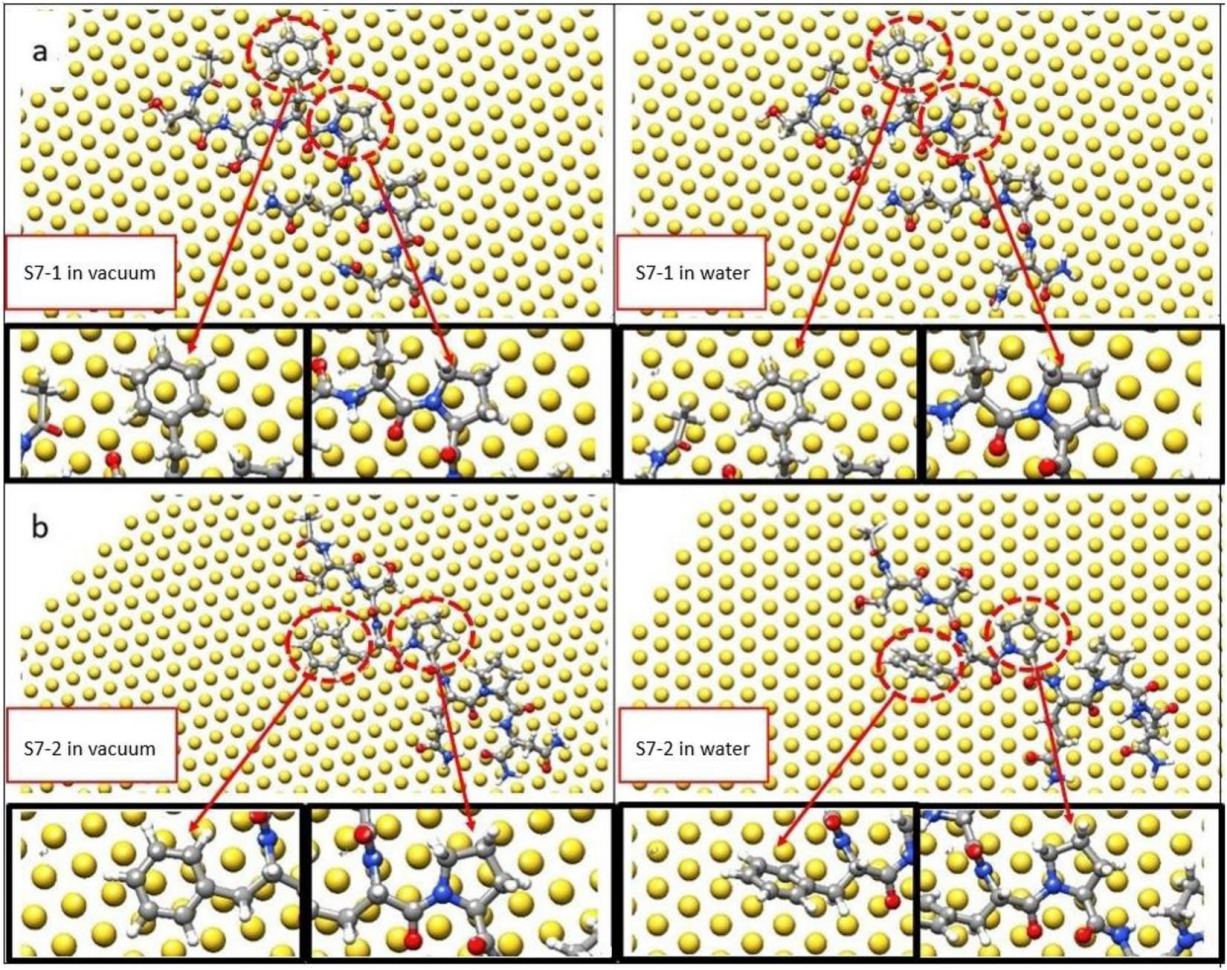
The stable adsorption configurations of S7-1 and S7-2 peptides in vacuum (left panels) and in water (right panels), with enlargements indicated by circles. (**a**) For S7-1, the six-membered Phe and the five-membered Pro are still stably locked to the Au(111) surface in the water environment. (**b**) For S7-2, locked six-membered ring of Phe in vacuum is not locked on the Au(111) in the water environment.

After the SABH search process in a vacuum, the two most stable S7 configurations on Au(111) were identified and are shown in the leftmost panels of Fig. 3(a,b). For ease in reading, these two cases of S7 adsorption will heretofore be designated as S7-1 in Fig. 3(a) and S7-2 in Fig. 3(b), and the binding energies of S7-1 and S7-2 on Au(111) in vacuum are listed in Table 2. The determined binding energy for S7-1 (−250.06 kcal/mol) was slightly higher than that of S7-2 (−252.84 kcal/mol) by approximately 1.1%. For S7-1 in the vacuum in the left panel, as shown in Fig. 3(a), the six-membered Phe ring and five-membered Pro ring connected by Phe were both locked by the Au(111) surface, as illustrated in the insets. For Phe on Au(111), a top Au atom was embedded by the Phe aromatic ring because three carbon atoms of aromatic ring match the second layer Au atoms and three remaining aromatic ring carbon atoms interact with the third Au atom layer. The hexagonal lattice can be seen on Au(111) surface, which is formed by the Au atoms in the second and third layers. It leads to the stronger van der Waals interactions between the carbon atoms of Phe aromatic ring and Au atoms directly below these carbon atoms, known as a lock-and-key (LAK) match^40^. Other than this LAK match of the Phe aromatic ring on Au(111), another LAK match can be seen between a specific orientated five-membered Pro ring on Au(111). The five-membered ring nitrogen atom occupies the top side of Au atom in the third layer while the two carbon atoms which bind the nitrogen atom then reside on top sites of the second layer Au atoms. The remaining two carbon atoms occupy the sites between the Au atoms in the first and third layers. Thus the Au(111) hexagonal lattice considerably enhances the strength of Van der Waals interactions between the five-membered Pro ring and Au(111) in the S7-1 orientation. For S7-2 in the vacuum, as shown in the leftmost panel in Fig. 3(b), only the Phe six-membered ring is stably locked by one top Au atom on the Au(111) surface. Compared to S7-1 then, the Au atom locking the Phe six-membered ring of S7-2 is not at the center of the ring, and the Pro five-membered ring is not stably locked by the Au(111), which is clear in the left panel inset in Fig. 3(b).

**Table 2:** Adsorption energies (Kcal/mol) for peptides S7-1 and S7-2 as on Au(111) in vacuum and in water.

	S7-1	S7-2
vacuum	−250.06	−252.84
water	−235.23	−210.26

The right panels of Fig. 3(a,b), respectively, show equilibrated S7-1 and S7-2 configurations in a water environment after MD simulation at temperature of 300 K for 5 ns. For S7-1, the six-membered Phe and the five-membered ring of Pro were stably locked by Au(111) in both vacuum and water environment as shown in Fig. 3(a), in which the six-membered ring and the five-membered ring make direct contact with the Au(111) by locking an Au top atom. However, the locked S7-2 six-membered ring of Phe in vacuum Fig. 3(b)(left) was not locked on Au(111), Fig. 3(b)(right). As shown in Table 2 for the water environment, the S7-1 binding energy with Au(111) is lower than that of S7-2 by approximately 11.89%. One can be seen that the S7-1 binding energy decreases from −250.06 to −235.23 kcal/mol and S7-2 binding energy decreased from −252.84 to −210.26 kcal/mol when the S7-1 and S7-2 adsorption system in the vacuum were changed to a water environment. For each STUN-BH iteration, the binding energy was calculated after the CG optimization and the peptide configuration on Au(111) is at its local minimum. In the water environment, the interaction between the peptide and water molecules cause a slight change in the peptide orientation on Au(111), weakening the binding energy in the water environment as compared to that in vacuum.

For S7-1 in Fig. 3(a), with local Phe and Pro LAK structures, it can see most S7-1 backbone atoms are also aligned in the same plane of six-membered Phe ring and five-membered Pro ring, leading to the stable S7 conformation on Au(111). Consequently, the conformation of S7-1 on Au(111) is unchanged during the 5 ns MD simulation, which can work as the perfect capping agent to protect the (111) surface of an AuNP. For S7-2 in the water environment, the S7-2 conformation continuously changes and slightly diffuses on Au(111) during the 5 ns MD simulation, indicating the S7-2 conformation on Au(111) is not a suitable conformation for the capping agent.

**Figure 4:**
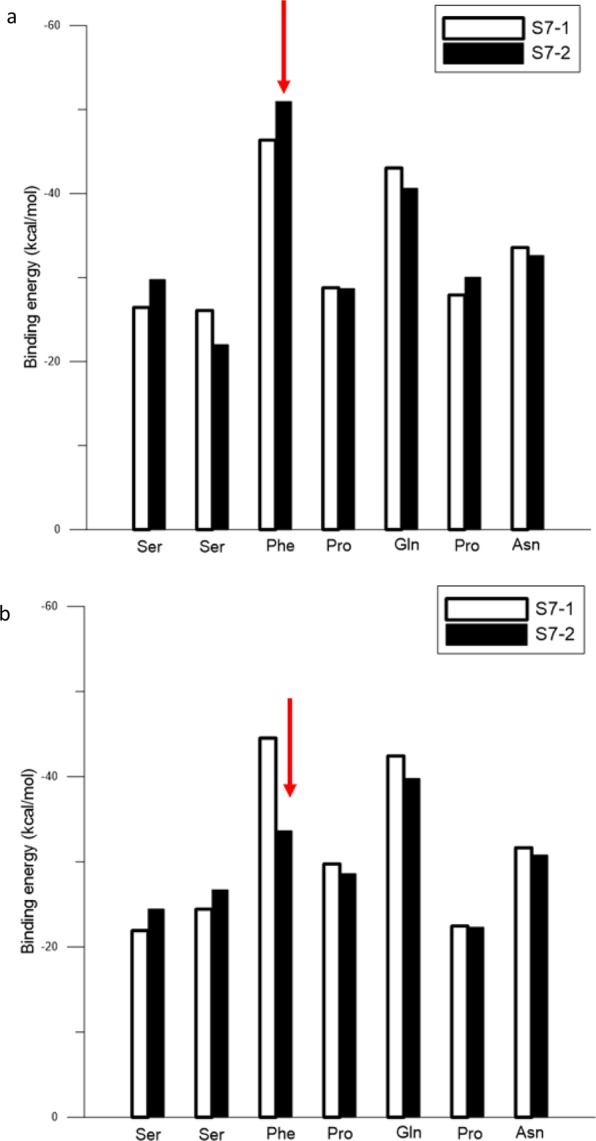
The binding energies for each amino acid residue for S7-1 and S7-2 with Au(111): (**a**) in vacuum and (**b**) in water.

Figure 4(a,b) show the respective binding energies of all amino acids with the Au(111) surface for S7-1 and S7-2 in vacuum and water. The amino acid sequence (from left to right) along the horizontal axis is the sequence from the S7 peptide N- to C-terminus. The binding energies of S7-2 Phe in the vacuum showed a distinct decrease from −51.0 to −33.75 kcal/mol when S7-2 was in the water. In Fig. 3(b) for S7-2, the flat-on Phe orientation on Au(111) (LAK match) in the vacuum did not remain while in the water environment. The Phe binding energy in vacuum exhibits a prominent decrease while in the water environment. For peptide S7-1, the binding energies in the vacuum were only slightly lower than those in water, because the LAK matches of Phe and Pro connected by the Phe of S7-1 can form the stable S7-1 configuration and orientation with Au(111) in the water environment.

The simulation results suggested that Phe with the six-membered ring and Pro with the five-membered ring were two critical amino acids in the S7 peptide forming a stable configuration on Au(111) and enhancing the growth of Au NPs with a higher fraction of the (111) facet. To verify the above finding, two peptides designated as PF8 and FS8 were designed, and their molecular structures and sequences were determined (Fig. 1(b,c), respectively). PF8 has three Pro-Phe pairs linked by Ser, while FS8 has four Phe amino acids linked by Ser. The Ser is used to make all Pro-Phe pairs of PF8 and all Phe residues of FS8 to exhibit greater flexibility on Au(111). If the Ser was not used for PF8 and FS8, the adsorption orientations of Pro-Phe pairs and Phe were influenced by the nearest Pro-Phe pairs and Phe, leading to less stable adsorption configurations on Au(111) than those with the Ser linkers for PF8 and FS8. The same STUN-BH process was carried out for both PF8 and FS8 in a vacuum, and then the most stable PF8 and FS8 configurations on Au(111) were relaxed by MD simulation in the water environment for 4 ns. For the STUN-BH results in the vacuum, all ring structures of PF8 and FS8 showed flat-on orientations to the Au(111) and were not observed again.

**Figure 5:**
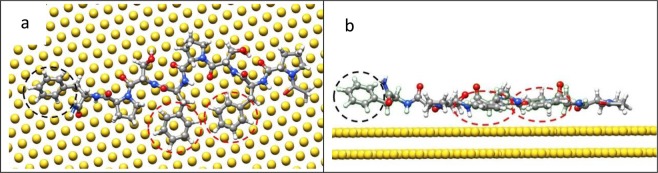
Representative snapshots of PF8 on the Au(111) surface in water: (**a**) top and (**b**) side views. The black circle shows the stand-up Phe, and red circles show the flat-on Phe. The water molecules are omitted due to diffusion out of the display region.

**Figure 6:**
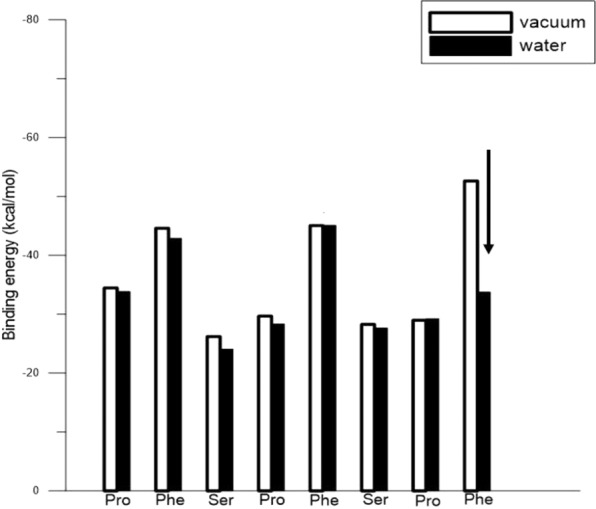
Binding energies of PF8 residues with Au(111) in vacuum and water.

Figure 5(a,b), respectively, show top and side views of relaxed PF8 configurations on Au(111) in the water environment. The six- and five-membered rings of two Phe-Pro pairs were still flat-on and are locked on the Au(111), while the Phe six-membered ring of at the C-terminus was in stand-up formation. Figure 6 shows the binding energies for PF8 residues with Au(111) in both vacuum and water. The binding energy for Phe at the C-terminus showed a distinct decrease (as indicated by the arrow) in water, while the binding energies for the remaining residues were only slightly weaker, indicating that PF8 can be used as a capping agent for AuNPs with the higher (111) facet.

**Figure 7:**
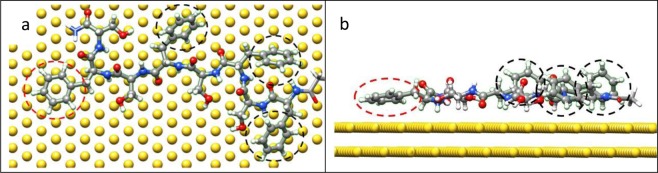
(**a**) Top and (**b**) side representative snapshots of FS8 on Au(111) surface in water environment. The black circle shows the stand-up Phe, and red circles show the flat-on Phe. The water molecules are not shown owing to diffusion out of the display region.

**Figure 8:**
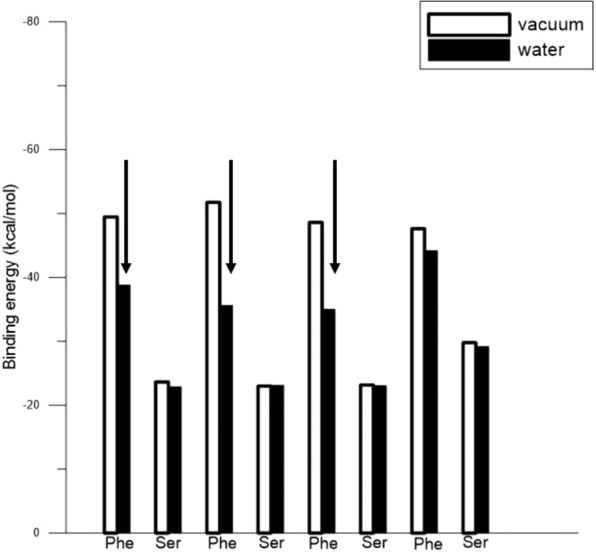
Binding energies of FS8 residues on Au(111) in vacuum and water.

Figure 7(a,b) show the top and side views of relaxed FS8 configurations on Au(111) in the water environment. One six-membered ring (as indicated by a red circle) of Phe was flat-on the Au(111) facet, and the other three six-membered rings (as indicated by black circles) of Phe residues showed stand-up orientations with the surface of the Au(111). Figure 8 details binding energies of FS8 residues on Au(111) in the vacuum and water. The binding energy decreases of the three Phe residues indicated by the black arrows were distinct, indicating that adsorption of FS8 on Au(111) in the water environment was less stable than that of PF8.

Our previous study^41^ used MD simulation to investigate the dynamic behavior of 1,3,5-triscarboxymethoxy benzene (TCMB, C_6_H_3_(OCH_2_COOH)_3_ on an Au(111) substrate at a temperature of 50 K. This study determined that, when LAK geometry appeared between the aromatic ring and Au(111) surface atom, the TCMB molecule resided atop the Au atom for 1.1–1.3 ns, which is significantly longer than for other orientations of the TCMB on the Au(111). In addition, for S7 and PF8 on Au (111), the local Phe-Pro pair LAK structures were predicted to extend the time that S7 and PF8 remained stably on the Au(111) facet, enhancing the growth of the (111) facet for AuNP. To verify the molecular simulation results suggesting that S7 and PF8 are appropriate capping agents for synthesizing AuNPs with the higher (111) facet fraction, corresponding experimental analysis was conducted. The AuNP synthesis with FS8 as the capping agent was also conducted to verify that FS8 was not stably adsorbed on Au(111) to enhance (111) facet growth.

### Characterization of synthesized NPs

**Figure 9:**
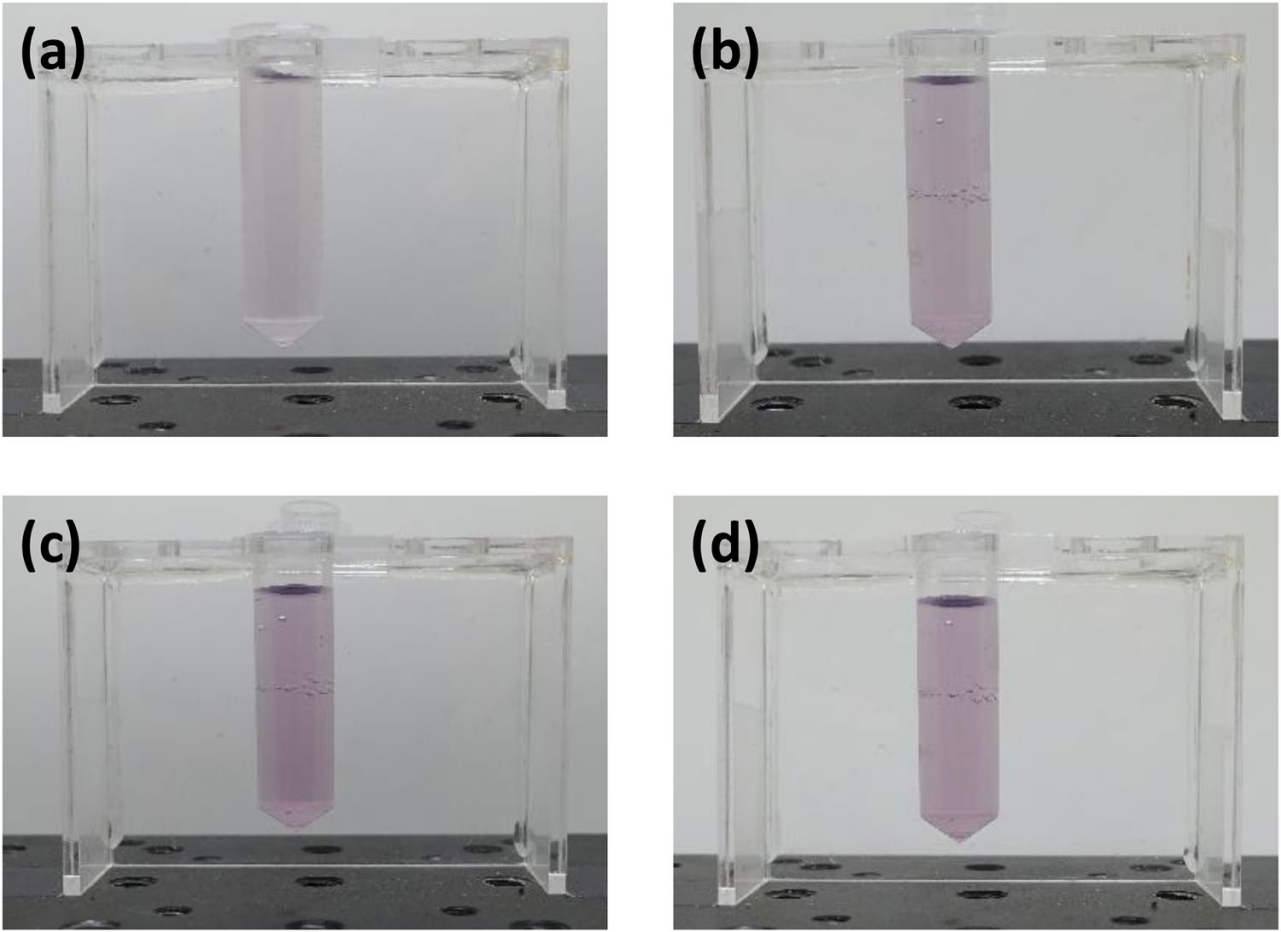
The photograph of the colloid gold solutions (**a**) right after (**b**) 24 hours, (**c**) 48 hours, and (**d**) 72 hours after the plasma treatment.

Figure 9 shows the time evolution of the color appearance of the synthesized AuNPs. The color became darker over time, demonstrating that Au^3+^ in the solution was reduced spontaneously. The synthesized metal NP was observed with a transmission electron microscope (TEM). The final colloid metal (100 μL) was diluted with 1900 μL of absolute alcohol (Echo Chemical, Tainan City, Taiwan). Next, 5 μL of the diluted colloid metal was pipetted onto a 200-mesh carbon-coated copper grid (FC200cu, EM Resolutions, South Yorkshire, UK), and was incubated statically for up to 24 h for volatilization of the solvent. TEM  speciemens were observed with an analytical scanning transmission electron microscope (JEM 3010, JOEL Ltd., Tokyo, Japan).

**Figure 10:**
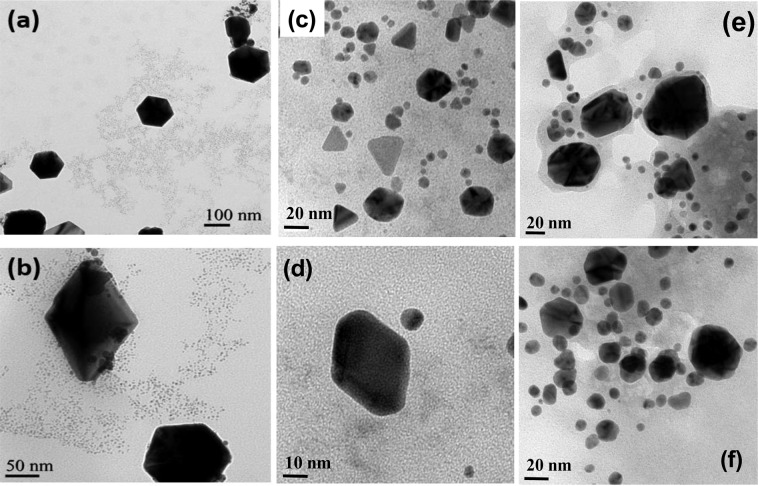
The TEM image of the gold nanoparticles capped by(**a**,**b**) S7, (**c**,**d**)PF8, (**e**,**f**) FS8.

Figure 10 shows the TEM images of AuNPs synthesized in the presence of S7, PF8, and FS8 peptides. The hexagonal- and diamond-shaped AuNPs corresponding to the (111) orientation were successfully synthesized in the presence of S7 peptide (Fig. 10a,b). This results confirmed that the AP plasma AuNP reduction and slow crystallization process were efficient for synthesizing specifically orientated AuNPs with the designed peptide, confirming that the S7 peptide was attached to the Au(111) surface of the gold crystal rather than on the Au(100) and Au(110) surface. Figures 10c,d present the TEM images for the AuNPs synthesized in the presence of PF8 peptides. The results also showed that the specific diamond and triangle shapes of the AuNPs corresponding to Au(111) plane-dependent face were synthesized. The designed PF8 peptide with the six-membered ring and neighboring 5-membered ring efficiently capped the Au(111) plane of the AuNPs while growing. Alternatively, AuNPs synthesized with the peptide without the five-membered ring for stabilizing the peptide capping on Au(111) plane showed arbitrary and round shapes. Although 4 six-membered rings were designed for the FS8 peptide, crystal-oriented AuNPs could not be formed because of the lack of a neighboring five-membered ring as the locking enhancement group.

Moreover, a significant “water-mark” was observed surrounding AuNPs synthesized in the presence of FS8 peptide, which was not observed for the AuNPs obtained using the S7 and PF8 peptides. Compared to the chemical structures of the S7 and PF8 peptides, the FS8 peptide exhibited a larger number of hydrophilic functional groups in the peptide chain such that FS8 peptide had a stronger affinity with water.

Additionally, the lack of the five-membered ring resulted in a weaker locking ability for FS8 peptide on the Au(111) plane; FS8 could not attach to the AuNP surface but was suspended in the solution because of its hydrophilicity. Similarly, the surface of plasma synthesized AuNPs with gold salt solution showed hydrophilic properties. The water-mark was formed after evaporating the water, leaving the water-dissolved peptide molecules surrounding the hydrophilic AuNPs. The experimental results confirmed that the co-existence of the 5-membered ring and 6-membered ring played an essential role in locking stability, supporting the results obtained using a molecular simulation approach.

## Conclusions

In this study, molecular simulation and experimental validation were conducted for the sequence design of a peptide capping agent on the gold (111) facet. The STUN-BH was used to identify the stable S7 peptide configuration on the Au(111) facet. The STUN-BH method and MD simulation were used to investigate the thermal stability between S7 and the Au facet at 300 K in both a vacuum and water environment. The CHARMM-METAL force field was used to model the adhesion force of S7, PF8, and FS8 peptides on the Au(111) surface. The simulation results indicated that the morphology of Pro affects the adsorption stability of Phe. Thus, the five-membered ring provided a particular geometry for peptide adsorption on the Au(111) plane and played an important role in peptide chain adsorption on the Au(111) plane. Two sequences of PF8 and FS8 peptides were designed and synthesized to validate the simulation results experimentally. The results showed that the FS8 peptide without the five-membered ring did not attach on the Au(111) plane and resulted in the formation of arbitrary and round-shaped AuNPs. The experimental results also confirmed that the presence of the five-membered ring significantly enhanced the absorption stability of the peptide chain on the Au(111) plane.

This study has provided a possible process to design the peptide sequence for the AuNP with the higher Au(111) facet by the STUN-BH method. In the related studies^17,19,20^, the CHARMM-METAL force field was used to study the S7 and T7 peptides on Pt(111) and Pt(100). The simulation results were expanded to the peptide sequence design for Au(111) in the current study. A reliable force field for accurately describing the interaction between the peptide and metal surface is necessary. For the Au surfaces, the GolP-CHARMM force field^42^ adopted a systematical parametrization process to obtain the reliable parameters for all amino acids and Au surfaces according to the experimental and first principles data. Consequently, the GolP-CHARMM force field could obtain more reliable results for different peptides on Au surface.
